# Recalcitrant Hailey-Hailey disease treated with isotretinoin: A family case series

**DOI:** 10.1016/j.jdin.2024.11.002

**Published:** 2024-11-24

**Authors:** Mahesh Mathur, Sandhya Regmi, Srijana Maharjan, Supriya Paudel, Nabita Bhattarai, Sambidha Karki

**Affiliations:** Department of Dermatology, College of Medical Sciences, Bharatpur, Nepal

**Keywords:** ATP2C1 mutation, familial benign chronic pemphigus, genodermatosis, Hailey-Hailey disease, isotretinoin

*To the Editor:* Hailey-Hailey disease (HHD) or familial benign chronic pemphigus is a rare, autosomal dominant genodermatosis characterized by vesicopustules, painful fissures, and erythematous plaques at sites of friction such as the neck, axilla, groin, and perineum.^1^^,^^2^ The gene implicated in the pathogenesis of HHD is ATP2C1 gene on chromosome 3q21-24, which encodes a calcium and manganese transporter of Golgi apparatus of cells.^1^^,^^2^ There is no cure for HHD; however, myriad modalities of treatment are reported in the literature with the main aim to control symptoms and reduce recurrence.^1^^,^^2^ We hereby report a family with recalcitrant HHD treated successfully with isotretinoin.

A family with 2 siblings, 60 years male (case 1) and 58 years male (case 2) presented with erythematous plaques with fissuring and maceration over nape of neck, bilateral axilla, groin, and perineum (Fig 1). The lesions started at the age of 35 years in case 1 and 51 years in case 2. Treatment with topical and oral antibiotics, antifungals, topical, and oral steroids failed to achieve sustained remission of dermatosis.

**Fig 1:**
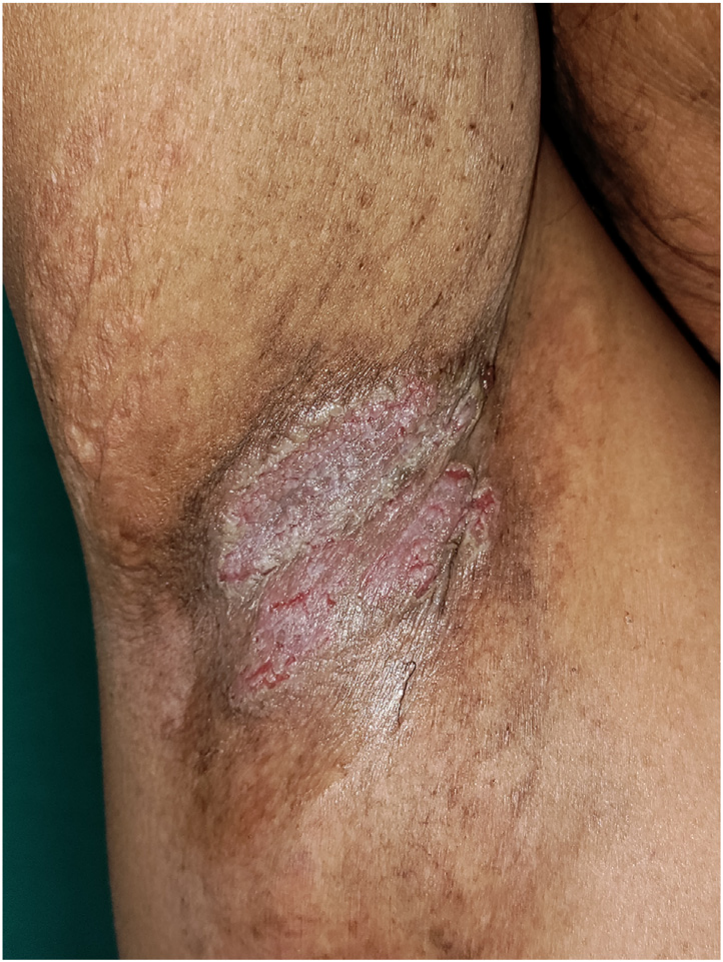
Hailey-Hailey disease. Ill-defined erythematous plaque with fissuring and maceration over right axilla.

Histopathology of skin biopsy showed intraepidermal clefting and suprabasal acantholysis resembling dilapidated brick wall appearance. Based on clinical and histopathologic findings, diagnosis of HHD was made and patients started with oral isotretinoin 0.5 mg/kg/d along with topical steroid and antibiotics.^3^ Significant improvement of lesions was seen in 6 weeks, after which topical medications were stopped and continued on isotretinoin monotherapy (Fig 2). A physical global assessment (PGA) scale was used to assess the efficacy of the treatment, with 0 indicating clear; 1, almost clear; 2, mild; 3, moderate; and 4, severe disease.^4^ The baseline PGA score was 4 in first case and 3 in second case, with rapid improvement (PGA 2 after 6 weeks of treatment) and sustained over time (PGA 1 at 3 and 0 at 6 months) in both cases. We aimed for total cumulative dose of 100 to 120 mg/kg of isotretinoin, which was achieved in 7 months, thereafter, we stopped the medication.^3^ The first case relapsed after 3 months of discontinuation of isotretinoin and was further controlled with topical steroids and antibiotics, whereas the second case has been in remission for the last 5 months during follow-up.

**Fig 2:**
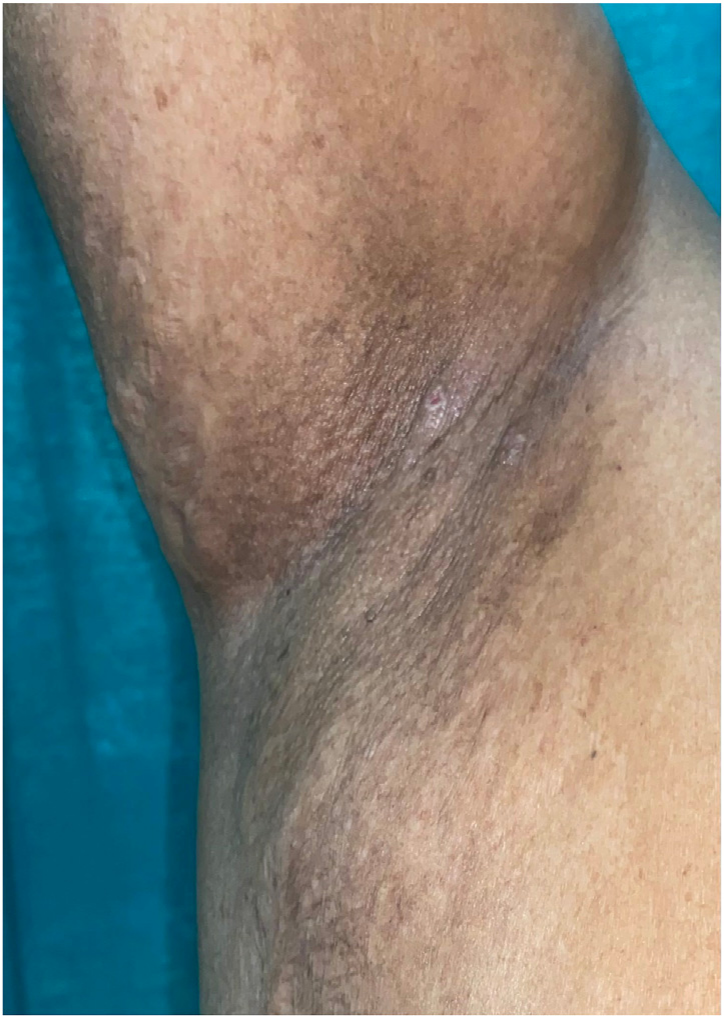
Hailey-Hailey disease postinflammatory hyperpigmentation over right axilla in 6-week follow-up after starting oral isotretinoin.

Isotretinoin, an 13-cis retinoic acid, in addition to being approved for acne, is used off-label in various skin conditions, including HHD.^2^^,^^3^ Although the mechanism of isotretinoin in HHD is not clearly defined, it may play a role in cell proliferation, differentiation, keratinization, cellular adhesiveness, immunomodulation and upregulation of ATP2CA1 gene expression.^5^ Multiple cases of HHD successfully treated with oral retinoids such as alitretinoin, etretinate, and acitretin are reported in the literature; however, there are only few case reports highlighting the role of isotretinoin in HHD.^1^^,^^2^^,^^5^ In our case series, 2 siblings responded well to isotretinoin, with maintenance of remission in 1 case and decrease in relapse frequency and severity in another case during postisotretinoin follow-up.

Our findings add to the nascent literature on the utility of oral isotretinoin for recalcitrant HHD; however, further controlled studies are needed to evaluate its efficacy.

## Conflicts of interest

None disclosed.
